# Parenting for Lifelong Health for Young Children: a randomized controlled trial of a parenting program in South Africa to prevent harsh parenting and child conduct problems

**DOI:** 10.1111/jcpp.13129

**Published:** 2019-09-19

**Authors:** Catherine L. Ward, Inge M. Wessels, Jamie M. Lachman, Judy Hutchings, Lucie D. Cluver, Reshma Kassanjee, Raymond Nhapi, Francesca Little, Frances Gardner

**Affiliations:** ^1^ Department of Psychology and Safety and Violence Initiative University of Cape Town Cape Town South Africa; ^2^ Department of Social Policy and Intervention University of Oxford Oxford UK; ^3^ MRC/CSO Social and Public Health Sciences Unit University of Glasgow Glasgow UK; ^4^ Centre for Evidence‐Based Early Intervention Bangor University Bangor UK; ^5^ Department of Psychiatry and Mental Health University of Cape Town Cape Town South Africa; ^6^ Department of Statistical Sciences University of Cape Town Cape Town South Africa; ^7^ Centre for Infectious Disease Epidemiology and Research (CIDER) School of Public Health and Family Medicine University of Cape Town Cape Town South Africa

**Keywords:** Parenting, Parenting for Lifelong Health, violence against children, low‐ and middle‐income countries, prevention

## Abstract

**Background:**

Parenting programs suitable for delivery at scale in low‐resource contexts are urgently needed. We conducted a randomized trial of Parenting for Lifelong Health (PLH) for Young Children, a low‐cost 12‐session program designed to increase positive parenting and reduce harsh parenting and conduct problems in children aged 2–9.

**Methods:**

Two hundred and ninety‐six caregivers, whose children showed clinical levels of conduct problems (Eyberg Child Behavior Inventory Problem Score, >15), were randomly assigned using a 1:1 ratio to intervention or control groups. At *t*_0_, and at 4–5 months (*t*_1_) and 17 months (*t*_2_) after randomization, research assistants blind to group assignment assessed (through caregiver self‐report and structured observation) 11 primary outcomes: positive parenting, harsh parenting, and child behavior; four secondary outcomes: parenting stress, caregiver depression, poor monitoring/supervision, and social support. Trial registration: ClinicalTrials.gov (NCT02165371); Pan African Clinical Trial Registry (PACTR201402000755243); Violence Prevention Trials Register (http://www.preventviolence.info/Trials?ID=24).

**Results:**

Caregivers attended on average 8.4 sessions. After adjustment for 30 comparisons, strongest results were as follows: at *t*_1_, frequency of self‐reported positive parenting strategies (10% higher in the intervention group, *p *=* *.003), observed positive parenting (39% higher in the intervention group, *p *=* *.003), and observed positive child behavior (11% higher in the intervention group, *p *=* *.003); at *t*_2,_ both observed positive parenting and observed positive child behavior were higher in the intervention group (24%, *p *=* *.003; and 17%, *p *=* *.003, respectively). Results with *p*‐values < .05 prior to adjustment were as follows: At *t*_1_, the intervention group self‐reported 11% fewer child problem behaviors, 20% fewer problems with implementing positive parenting strategies, and less physical and psychological discipline (28% and 14% less, respectively). There were indications that caregivers reported 20% less depression but 7% more parenting stress at *t*_1_. Group differences were nonsignificant for observed negative child behavior, and caregiver‐reported child behavior, poor monitoring or supervision, and caregiver social support.

**Conclusions:**

PLH for Young Children shows promise for increasing positive parenting and reducing harsh parenting.

## Abstract 2

Read the Commentary on this article at doi: 10.1111/jcpp.13172

## Introduction

Parenting programs have been identified as a key strategy for preventing violence against children. They are thus critical to achieving United Nations SDG 16.2, to end “all forms of violence against children” (WHO, 2016). Delivered as early interventions, they are effective in reducing child conduct problems and youth risk behaviors (Piquero, Farrington, Welsh, Tremblay, & Jennings, 2009).

Effectiveness of parenting programs is well established (e.g., Chen & Chan, 2015; Knerr, Gardner, & Cluver, 2013), but questions remain about the best targeting and delivery strategies for achieving SDGs. Globally, violence against children is more prevalent in LMIC (Hillis, Mercy, Amobi, & Kress, 2016), as are conditions that increase parenting difficulties, including poverty and related stressors such as community violence. Yet, evidence comes chiefly from HIC (Chen & Chan, 2015; Knerr et al., 2013; Leijten, Melendez‐Torres, Knerr, & Gardner, 2016), and many evidence‐based parenting programs are costly and culturally Western; these factors, particularly cost factors, may make them inappropriate for low‐resource LMIC contexts, especially for scale‐up (Mikton, 2012).

With these issues in mind, we developed PLH for Young Children, a low‐cost parenting program for caregivers of children aged 2–9 (Lachman, Sherr et al., 2016). Program development involved integrating evidence and content from HIC's (e.g., Hutchings, 2013) with findings from a formative evaluation with South African caregivers and service providers (Lachman, Cluver et al., 2016). Several principles guided development: evidence for effective components based on social learning principles (Hutchings, Gardner, & Lane, 2004) and the need to train parent group facilitators to work collaboratively with caregivers (Eames et al., 2009; Furlong et al., 2013). Mindfulness‐based stress reduction exercises were included to address caregiver‐identified needs (Lachman, Sherr, et al., 2016). The program also included traditional Southern African stories, songs, and experiential activities to increase its cultural acceptability. For affordability, the program was designed to be delivered by lay community members. Materials were kept low‐cost, easily adaptable, and suitable for low literacy contexts.

The program was tested in a pilot RCT, which suggested that although the program had promise, revisions might strengthen its impact and feasibility (Lachman, Cluver, et al., 2016; Lachman et al., 2017). Content on positive reinforcement and discipline was subsequently refined, and additional training was provided to facilitators to strengthen competency in the collaborative process and in understanding social learning theory.

The revised program was the subject of this larger RCT, with the objective of exploring whether a program designed for the conditions of LMIC could be delivered with fidelity, acceptable to caregivers, and effective in increasing positive parenting and decreasing harsh parenting, thereby reducing child conduct problems. We aimed to target families at elevated risk for harsh parenting by screening for the presence of parental concern about child conduct problems (Piquero et al., 2009). We designed the trial with scale‐up in mind: The program was tested under conditions likely to prevail in South Africa, and within a local “real‐world” service, an NGO. We assessed outcomes immediately postintervention, and one year later, and used observational assessments of caregiver–child interaction to address potential bias in self‐report measures.

## Method

### Setting

The study was conducted between February 2014 and March 2016 in two historically black African peri‐urban settlements, among the most deprived in Cape Town, with high levels of HIV and community and family violence.

### The program

Facilitators were paraprofessional community members with high school level education who were hired and trained during the first pilot study to conduct the program (Lachman et al., 2017).

First, facilitators visited each family at home to explore caregivers’ goals for their children and discuss any questions they had. Drawing on principles common to many evidence‐based parenting programmes, the first half of the program focused on positive relationship building through dedicated one‐on‐one time and positive reinforcement of desirable behaviour. Subsequent sessions taught limit‐setting through instruction giving, household rules, and daily routines; and nonviolent discipline strategies using redirect, ignore, time‐out, and consequences for decreasing undesirable behavior. Caregivers practiced new skills in role‐play during each of the 12 three‐hour sessions and at home with their children. They reported to the group on their home practice, with facilitators underlining the principles of effective parenting through modeling praise and leading group problem‐solving to resolve challenges. For full program manual, see http://www.who.int/violence_injury_prevention/violence/child/plh/en/.

### Participants

**Figure 1:**
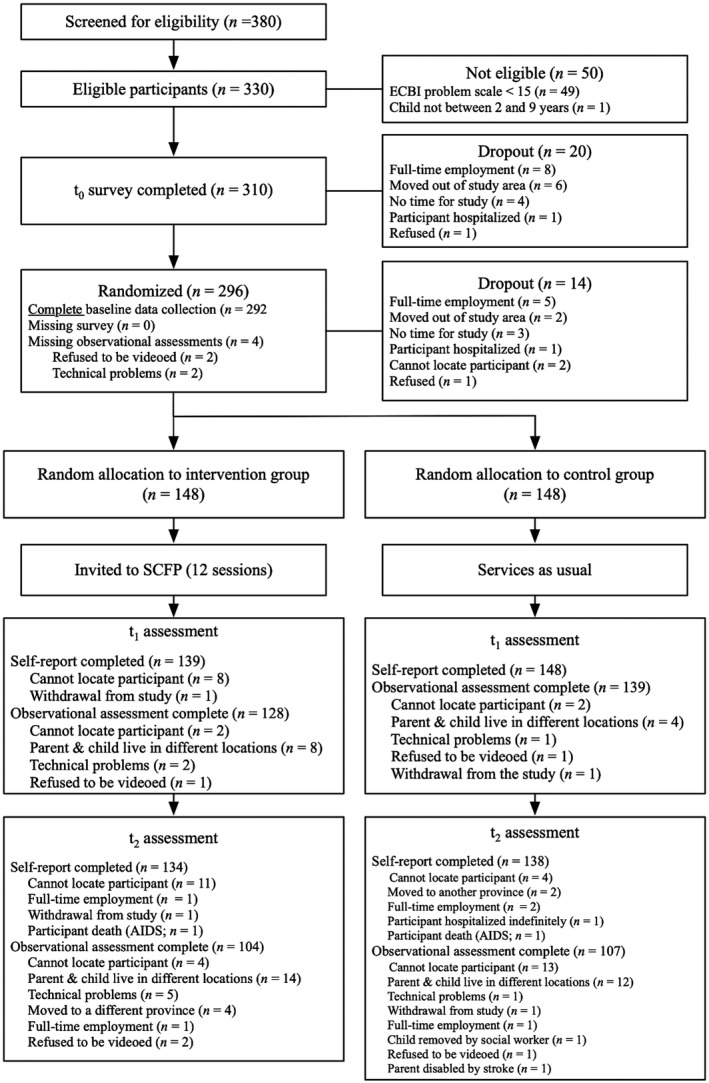
CONSORT flow diagram

Through targeted sampling and referrals from local agencies, 380 child–caregiver dyads were recruited and screened for trial eligibility. Inclusion criteria for adults included: age 18 + years; primary caregiver of child aged 2–9 years, regardless of status as biological parent; coresiding with child 4 + nights per week; and reporting 15 + problem behaviors on the ECBI problem scale. Of 330 eligible, consenting parents, 310 completed the baseline survey (Figure 1) and 296 were subsequently randomized to intervention or control arm in a 1:1 ratio. Stratified randomization ensured a balanced design with respect to child age (2‐ to 5‐year‐olds and 6‐ to 9‐year‐olds) and sex, within each community.

Two participants were referred by schools and 18 by child welfare organizations. The majority of participants (*n *=* *360) were approached through researchers going door‐to‐door to every eighth home in the communities, after initial attempts to recruit via agencies did not yield sufficient participants. Caregivers were invited to identify one child aged 2–9 on whom to focus for eligibility screening.

All caregivers gave informed consent to participate. Ethical approval was granted by the Research Ethics Committee, Faculty of Humanities, University of Cape Town (Ref: PSY2014‐001), and the Inter‐Divisional Research Ethics Committee Oxford University (Ref: SSD/CUREC2/11‐40).

### Measures

Parenting and child behavior were the primary outcomes, assessed from multiple perspectives. For self‐reported parenting, we used Setting Limits and Supporting Positive Behavior subscales of the Parenting Young Children Scale (PARYC; McEachern et al., 2011), grouped together as “Positive Parenting.” Nonviolent discipline, and psychological and physical punishment, were assessed using the ICAST‐Parent Report (Runyan et al., 2009), adapted for trial use by asking only about the past month. Child behavior in the past month was assessed using the ECBI (Eyberg & Ross, 1978) intensity and problem scales. Caregivers and children also participated in a structured observational task: Research assistants asked caregivers to play with their children for ten minutes and then to ask the child to return the toys to the research assistant. This was video‐recorded and later coded using a simplified version of the Dyadic Parent–Child Interaction Coding System (Robinson & Eyberg, 1981). Caregiver behaviors were coded either as positive (e.g., smiles) or negative (e.g., criticizing), as were child behaviors: negative (e.g., being rude to caregiver) and positive (e.g., affection). Inter‐rater reliability was assessed by trained research assistants, on a subsample of 40% of the videos, achieving above *κ = *.7 for all codes.

Secondary outcomes included parenting stress (Parenting Stress Index – Short Form; Abidin, 1997); depression (Beck Depression Inventory–II; Beck & Steer, 1988); exposure to IPV (Revised Conflict Tactics Scale; Straus, Hamby, Boney‐McCoy, & Sugarman, 1996); poor monitoring and supervision (Alabama Parenting Scale; Essau, Sasagawa, & Frick, 2006); and social support (Medical Outcomes Study Social Support Survey; Sherbourne & Stewart, 1991). Substance misuse was assessed as follows: for alcohol misuse, with one question validated in South Africa (Mertens, Ward, Bresick, Broder, & Weisner, 2014) for distinguishing problem from low‐risk drinkers; for other drugs, use in the last month. For a specific list of primary and secondary outcomes, see Table 3.

Demographic variables assessed at baseline included caregiver age, sex, level of education, marital status, employment status, and household income source; child age, sex, school attendance, and relationship to caregiver; household poverty (Hunger Scale; Labadarios et al., 2005); caregiver's own history of maltreatment as a child (ICAST – Retrospective; Dunne et al., 2009); HIV status of caregiver and child (self‐report); and child's orphan status.

Implementation fidelity was assessed using facilitator completed postsession checklists of intervention activities and by videotaping group sessions. Videos were used to verify facilitator checklists, and in supervision to assist facilitator skill and fidelity. Caregiver attendance was recorded. Since the program allowed for home‐visit consultations if a caregiver was unable to attend a session, caregivers were counted as attending the session if a home visit was successfully completed. Program acceptability was assessed using a 40‐item questionnaire adapted from the Incredible Years Parent Satisfaction Questionnaire (http://www.incredibleyears.com/download/Evaluations/Final_Parent_Satisfaction_Questionnaire112013.pdf).

All measures were translated into isiXhosa (the local language) by consensus forward translation and checked by back translation.

### Procedures

Baseline data were collected during March and April 2014 in the first community and from late June to mid‐August 2014 for the second. Randomization was conducted after data collection by an off‐site statistician with no other contact with the trial. Facilitators informed participants of their allocation to intervention or control. The program began 4 weeks after randomization (*t*_0_) in both communities. The immediate post‐test (*t*_1_) began 17.5 weeks after randomization in the first community and 20 weeks postrandomization (to accommodate summer holidays) in the second. The 1‐year follow‐up (*t*_2_) data were collected 70 and 71 weeks postrandomization in the first and second communities, respectively.

Data were collected in participants’ homes. Researchers were accompanied by community guides, and before researchers entered the home, community guides first requested caregivers to keep their allocation confidential. Care was thus taken to keep researchers as blind to allocation as possible, although inadvertent disclosure remained possible.

### Data analysis

The analysis plan was developed in advance of analysis. All analyses were conducted in R, version 3.4.3 (R Core Team, 2017). Internal consistency of each measure was assessed using reliability coefficients Cronbach's alpha, omega, and greatest lower bound. Outcome measures were summarized by arm and time point (mean and standard deviations; and median and first and third quartiles).

Each outcome was assessed through several composite scores, derived by summing either Likert scale assessment of the intensity of a behavior or binary indicators of the presence of a trait. The underlying distributions of the composite scores were modeled assuming either Gaussian (for sums of many individual items with symmetric empirical distributions), Negative Binomial (for over‐dispersed count outcomes), or Poisson (for count outcomes) distributions. A log link was used for all models, thus providing multiplicative differences (interpretable as percentage differences) between groups.

The study design imposed within‐subject and within‐group correlation. Multilevel generalized linear models were used to compare the intervention and control arms with respect to changes in behavioral outcomes over time. The models included a time effect (comparison of outcomes at immediate post‐test and 1‐year follow‐up, respectively, to outcomes at baseline) and allowed for a modification of the time effect due to the intervention (through the inclusion of interaction terms), while capturing both that the same child–caregiver dyads were assessed at *t*_0_, *t*_1_, and *t*_2_, and the group‐based nature of the intervention through the inclusion of dyad‐specific and group‐specific random effects. The models also included adjustment for randomization stratifier child gender, child age, and community, and followed an intent‐to‐treat approach.

The size of intervention effects at *t*_1_ and *t*_2_, compared with *t*_0_, was estimated by the exponents of the coefficients of the interaction terms (due to the use of the log links), which measured the proportional difference in the change from baseline in the outcomes at *t*_1_ and *t*_2_ for the intervention group compared with the control group. Observed *p*‐values are reported throughout the paper for all tests carried out, as a means of assessing strength of association rather than using a threshold value to determine statistical significance. Lower *p*‐values are indicative of stronger associations and larger ones of weaker or no meaningful association. Additionally, should the focus be on hypothesis testing, Holm's method was used to calculate adjusted *p*‐values that control the family‐wise error rate taking into account the focus on two effect sizes for each of 15 outcomes (i.e., 30 comparisons).

Post hoc power calculations were based on effect sizes as measured by the beta coefficients for the interaction terms in the generalized linear mixed models, assuming a variability in effect sizes of .55 (calculated as average of standard error of beta times the square root of sample size). The randomized sample sizes of 148 per arm allowed for detection of a minimum proportional difference between the intervention and control group with respect to changes from baseline of 22% with 90% power if a significance level of 5% was chosen, taking into account the (30) multiple comparisons. The detectable proportional difference becomes 27% at a minimum sample size of 104 per arm – accounting for the outcome with the most missing data. Smaller effects may be identifiable if they are associated with smaller variability.

### Trial registration

The trial was registered at ClinicalTrials.gov (NCT02165371), the Pan African Clinical Trial Registry (PACTR201402000755243), and the Violence Prevention Trials Register (http://www.preventviolence.info/Trials?ID=24).

## Results

### Sample characteristics

Most (240, 81.1%) caregivers were the child's biological mother. One male caregiver was recruited, allocated to the intervention group, and did not attend but completed all assessments. The groups were similar in terms of most characteristics (see Table 1), with the apparent exceptions that the intervention arm had more HIV‐positive caregivers and more who reported IPV, while the control group had slightly more employed caregivers. Household vulnerability is illustrated by 26 children (8.8%) having lost at least one caregiver; 76 (25.7%) caregivers reporting being HIV‐positive, 93 (31.4%) reporting risky alcohol use, 89 (30.1%) reporting past month IPV; and 140 (47.3%) reporting physical, 98 (33.1%) emotional, and 19 (6.4%) sexual abuse as children. Most caregivers (253, 85.5%) were unemployed.

**Table 1:** Characteristics of the sample, by study arm, at baseline

Characteristic	Category	Frequency (%)	
		Control	Intervention
Child age group	2–5	79 (53.38)	82 (55.41)
	6–9	69 (46.62)	66 (44.59)
Child sex	Female	69 (46.62)	69 (46.62)
Caregiver age	18–24	24 (16.22)	23 (15.54)
	25–39	87 (58.78)	92 (62.16)
	40–59	32 (21.62)	30 (20.27)
	60+	5 (3.38)	3 (2.03)
Caregiver sex	Female	147 (99.32)	148 (100.00)
Relationship to child	Step‐sibling	1 (0.68)	2 (1.35)
	Aunt/uncle	7 (4.73)	8 (5.41)
	Grandparent	23 (15.54)	15 (10.14)
	Mother	117 (79.05)	123 (83.11)
Currently working	No	123 (83.11)	130 (87.84)
HIV status of caregiver	Positive	30 (20.27)	46 (31.08)
	Unknown	8 (5.41)	5 (3.38)
HIV status of child	Positive	1 (0.68)	2 (1.35)
	Unknown	73 (49.32)	59 (39.86)
Past month alcohol abuse	Yes	44 (29.73)	49 (33.11)
	Unknown	1 (0.68)	0 (0.00)
Past month drug abuse	Yes	8 (5.41)	11 (7.43)
Orphan status	Double	1 (0.68)	0 (0.00)
	Single	12 (8.11)	13 (8.78)
	Unknown	3 (2.03)	2 (1.35)
Past month IPV	Yes	38 (25.68)	51 (34.46)
	Unknown	1 (0.68)	2 (1.35)
Childhood physical abuse	Yes	67 (45.27)	73 (49.32)
	Unknown	19 (12.84)	10 (6.76)
Childhood emotional abuse	Yes	47 (31.76)	51 (34.46)
	Unknown	21 (14.19)	13 (8.78)
Childhood sexual abuse	Yes	9 (6.08)	10 (6.76)
	Unknown	7 (4.73)	8 (5.41)

All scales (see Appendix S1 ) had alpha, omega or greatest lower bound .7 or greater, except for the ICAST physical and nonviolent discipline subscales (probably because of skewness and zero‐inflation; Trizano‐Hermoslia & Alvarago, 2016).

For caregiver self‐report, the follow‐up rate was 97.0% at *t*_1_ and 91.9% at *t*_2_. Follow‐up rates for observations were lower (90.2% at *t*_1_; 71.3% at *t*_2_; see Figure 1). Those lost to follow‐up were more likely to report drug use (13.3% vs. 5.6%), and physical abuse as a child (58.3% vs. 51.8%), and slightly less likely to report use of nonviolent discipline of their own child (mean 5.9 vs. 6.4). They were observed to show more positive (mean 16.9 vs. 13.6) and negative (mean 3.7 vs. 2.9) interactions with their child; their children showed more negative behaviors (mean 58.6 vs. 48.3). These were small differences but may suggest a slightly more negative profile for parents who were lost to follow‐up.

### Program implementation and acceptability

Facilitators delivered 96.8% of the manualized activities. All parenting skill components were delivered; activities that were not covered were less central, such as “energizer” exercises. Most (110, 74.3%) caregivers attended at least one group session, and average overall attendance (group sessions plus home visits) was 8.4 sessions (70%), figures which are within the range for other parenting programs (Chacko et al., 2016). The 84 caregivers (56.8%) who attended the last session (#12) reported very high overall program satisfaction (*M *=* *94.9%; *SD *=* *8.2).

### Program effects

Table 2 presents data at all three time points and Table 3 the results of GLMMs for intervention and control group differences. Both groups improved over time, but there were a number of differences between groups.

**Table 2:** Outcome variables, by study arm, by three time points^a^

Outcome	Arm	Mean (*SD*)			Median (quartile 1, quartile 3)		
		*t*_0_	*t*_1_	*t*_2_	*t*_0_	*t*_1_	*t*_2_
ECBI: intensity score	Control	143.00 (23.14)	115.64 (27.50)	104.16 (26.81)	142.50 (126.00, 157.25)	116.00 (93.00, 133.00)	103.00 (84.00, 122.00)
	Intervention	141.21 (22.85)	111.58 (24.82)	100.59 (26.64)	142.00 (125.75, 155.25)	113.00 (94.00, 130.00)	98.00 (80.00, 117.00)
ECBI: problem score	Control	25.16 (4.94)	18.03 (8.46)	13.01 (8.73)	26.00 (21.00, 28.00)	18.50 (11.75, 24.00)	12.00 (6.00, 19.00)
	Intervention	24.61 (5.07)	16.05 (7.93)	12.78 (8.48)	25.00 (20.00, 29.00)	16.00 (10.00, 22.00)	11.00 (6.00, 17.00)
Positive Parenting: frequency score	Control	49.23 (11.42)	49.88 (11.33)	53.62 (8.99)	52.00 (44.00, 56.00)	51.00 (43.50, 58.00)	54.00 (49.00, 59.00)
	Intervention	47.55 (9.31)	54.86 (9.37)	53.05 (9.99)	48.00 (42.00, 53.00)	56.00 (50.50, 59.50)	54.00 (48.00, 60.00)
Positive Parenting: problem score	Control	4.78 (3.96)	3.66 (3.78)	1.76 (2.41)	4.00 (1.00, 8.00)	2.00 (1.00, 6.00)	1.00 (0.00, 3.00)
	Intervention	4.38 (3.36)	2.76 (3.31)	1.89 (2.75)	4.00 (2.00, 6.00)	1.00 (1.00, 4.00)	1.00 (0.00, 3.00)
Nonviolent Discipline	Control	6.34 (3.29)	5.18 (2.94)	4.21 (2.46)	6.00 (4.00, 9.00)	5.00 (3.00, 7.00)	4.00 (2.00, 6.00)
	Intervention	6.42 (3.24)	5.43 (2.93)	4.92 (2.93)	6.00 (5.00, 8.50)	5.00 (3.00, 7.00)	5.00 (3.00, 7.00)
Physical Discipline	Control	4.67 (4.19)	2.77 (3.25)	1.50 (1.94)	4.00 (2.00, 7.00)	2.00 (0.00, 4.00)	1.00 (0.00, 2.00)
	Intervention	4.22 (3.47)	1.99 (2.83)	1.49 (2.27)	4.00 (2.00, 6.00)	1.00 (0.00, 3.00)	0.00 (0.00, 2.00)
Psychological Discipline	Control	6.91 (5.39)	3.77 (3.99)	2.45 (2.97)	5.00 (3.00, 10.00)	3.00 (1.00, 5.00)	2.00 (0.00, 4.00)
	Intervention	6.89 (4.62)	3.19 (3.38)	2.49 (2.50)	6.00 (3.00, 9.25)	2.00 (1.00, 5.00)	2.00 (0.00, 4.00)
Observed Positive Parenting	Control	12.63 (10.32)	8.79 (8.45)	6.79 (6.62)	10.00 (5.00, 16.00)	6.00 (3.50, 12.00)	5.00 (2.00, 9.50)
	Intervention	15.27 (15.54)	14.23 (14.95)	10.83 (15.57)	11.00 (5.00, 22.00)	10.00 (4.00, 20.00)	6.00 (2.00, 14.00)
Observed Positive Child Behavior	Control	27.94 (23.94)	24.11 (21.17)	16.39 (14.90)	20.00 (9.00, 47.00)	20.00 (7.50, 33.00)	12.00 (5.00, 23.00)
	Intervention	26.76 (20.07)	26.10 (22.57)	18.57 (18.55)	22.00 (10.00, 41.00)	18.00 (11.00, 35.00)	11.00 (6.00, 24.25)
Observed Negative Parenting	Control	2.86 (3.37)	2.49 (3.73)	2.49 (5.74)	2.00 (0.00, 4.00)	1.00 (0.00, 3.00)	1.00 (0.00, 3.00)
	Intervention	3.14 (4.59)	2.67 (3.52)	1.88 (2.84)	2.00 (1.00, 4.00)	2.00 (0.00, 3.00)	1.00 (0.00, 2.00)
Observed Negative Child Behavior	Control	1.75 (2.92)	1.42 (3.17)	0.60 (1.64)	0.00 (0.00, 2.00)	0.00 (0.00, 1.00)	0.00 (0.00, 0.00)
	Intervention	1.71 (3.98)	1.51 (2.33)	0.68 (1.50)	0.00 (0.00, 2.00)	0.50 (0.00, 2.00)	0.00 (0.00, 1.00)
Caregiver Depression	Control	15.39 (12.12)	10.90 (10.48)	8.41 (10.62)	12.50 (6.00, 23.25)	7.00 (3.00, 15.25)	3.00 (0.00, 15.00)
	Intervention	15.74 (10.90)	8.75 (8.44)	7.75 (9.26)	14.50 (6.00, 24.00)	6.00 (2.00, 13.00)	4.00 (1.00, 12.00)
Parenting Stress	Control	111.92 (21.53)	123.12 (21.22)	130.25 (15.76)	116.00 (100.25, 126.00)	129.50 (114.00, 138.00)	133.00 (122.00, 140.00)
	Intervention	114.59 (19.33)	127.56 (21.16)	133.42 (13.20)	115.00 (103.50, 126.00)	134.00 (119.00, 141.00)	135.00 (127.00, 143.00)
Poor Monitoring and Supervision	Control	22.03 (4.60)	21.06 (4.08)	20.01 (3.65)	21.00 (19.00, 25.00)	21.00 (18.00, 24.00)	20.00 (18.00, 23.00)
	Intervention	21.99 (4.53)	21.85 (3.82)	20.19 (3.27)	22.00 (18.00, 24.75)	21.00 (19.00, 24.00)	20.00 (17.00, 22.00)
Caregiver Social Support	Control	20.58 (6.29)	20.60 (6.57)	19.41 (6.68)	21.00 (16.00, 24.00)	20.50 (16.00, 24.75)	20.00 (14.00, 24.00)
	Intervention	21.03 (5.98)	20.51 (6.02)	19.60 (6.53)	22.00 (17.00, 24.00)	20.00 (18.00, 23.00)	21.00 (15.00, 24.00)

a Sample sizes are 132–148 (baseline), 128–148 (immediate post‐test), 104–137 (one‐year follow‐up).

**Table 3:** Proportional change in the intervention group compared with the control group, from baseline^a^

Outcome	Comparing *t*_1_ to *t*_0_			Comparing *t*_2_ to *t*_0_			Distribution
	Ratio^b^ (95% CI)	Unadjusted *p*‐value	Holm's adjusted *p*‐value^c^	Ratio^b^ (95% CI)	Unadjusted *p*‐value	Holm's adjusted *p*‐value^c^	
Primary outcomes							
ECBI: intensity score	0.9636 (0.9152–1.0147)	.1592	1.000	0.9645 (0.9092–1.0232)	.2296	1.0000	Gaussian
ECBI: problem score	0.8897 (0.8043–0.9841)	.0232	.534	0.9833 (0.8578–1.1271)	.8082	1.0000	Gaussian
Positive Parenting: frequency	1.1015 (1.0534–1.1518)	*<*.0001	.003	0.9897 (0.9461–1.0352)	.6498	1.0000	Gaussian
Positive Parenting: problem	0.8036 (0.6807–0.9486)	.0100	.250	1.0719 (0.8707–1.3195)	.5131	1.0000	Negative Binomial
Nonviolent Discipline	1.0397 (0.9397–1.1502)	.4508	1.000	1.1669 (1.0432–1.3053)	.0071	.199	Negative Binomial
Physical Discipline	0.7230 (0.5821–0.8981)	.0035	.091	0.9583 (0.7451–1.2325)	.7401	1.0000	Negative Binomial
Psychological Discipline	0.8642 (0.7354–1.0157)	.0770	1.000	1.0516 (0.8744–1.2646)	.5933	1.0000	Negative Binomial
Observed Positive Parenting	1.3914 (1.2940–1.4962)	<.0001	.003	1.2410 (1.1301–1.3628)	*<*.0001	.003	Poisson
Observed Positive Child Behavior	1.1098 (1.0578–1.1644)	<.0001	.003	1.1675 (1.0943–1.2456)	<.0001	.003	Poisson
Observed Negative Parenting	1.0353 (0.8909–1.12031)	.6510	1.000	0.8330 (0.6923–1.0022)	.0528	1.000	Poisson
Observed Negative Child Behavior	1.1003 (0.9019–1.3425)	.3462	1.000	1.2009 (0.8558–1.6852)	.2895	1.000	Poisson
Secondary outcomes:							
Caregiver Depression	0.7990 (0.6675–0.9565)	.0148	.355	0.9343 (0.7752–1.1260)	.4756	1.0000	Negative Binomial
Caregiver Social Support	0.9949 (0.9258–1.0691)	.8881	1.000	1.0088 (0.9335–1.0901)	.8250	1.0000	Gaussian
Poor Monitoring and Supervision	1.0391 (0.9881–1.0929)	.1359	1.000	1.0132 (0.9608–1.0685)	.6289	1.0000	Negative Binomial
Parenting Stress (reversed)^d^	0.9276 (0.8832–0.9742)	.0028	.076	0.9576 (0.9098–1.0079)	.0976	.5856	Negative Binomial

a Models controlled for child age and sex, and community.

b Ratio of change from *t*_0_ to *t*_1_ or *t*_2_ in intervention group to change from *t*_0_ to *t*_1_ or *t*_2_ in control group as estimated by exp(ß) for the interaction effect between group and time components.

c Adjusted for 30 comparisons, to account for two comparisons for each of 15 outcomes.

d Parenting Stress was reversed to improve model fit; decreased scores imply increase in parenting stress.

At *t*_1_, the strongest differences (based on both the original and the adjusted *p*‐values) between the intervention and control groups were observed with respect to the frequency of self‐reported positive parenting strategies (10% higher in the intervention group), observed positive parenting (39% higher), and observed positive child behavior (11% higher). Intervention impact on observed positive parenting and positive child behavior endured at *t*_2_, with higher frequencies of 24% and 17%, respectively. Additionally, based on the unadjusted *p*‐values, at *t*_1_ there were indications that the intervention group self‐reported fewer child problem behaviors (11% fewer), had fewer problems with implementation of positive parenting strategies (20% fewer), and self‐reported less physical and psychological discipline (28% and 14% less, respectively). Likewise, there were indications that caregivers reported less depression (20% less) but more parenting stress at post‐test (7% more). Differences at either *t*_1_ or *t*_2_ in caregiver‐reported child behavior, observed negative child behavior, poor monitoring or supervision, or caregiver social support were much smaller or negligible as confirmed by smaller effect sizes and larger *p*‐values.

## Discussion

To be suitable for scale‐up, a program must demonstrate effectiveness, be delivered with fidelity, and be acceptable to caregivers (Gottfredson et al., 2015). PLH for Young Children was specifically designed to be suitable for, and was tested under, the conditions that prevail in LMIC.

Effects on primary outcomes included increases in observed positive parent and child behavior, and, at *t*_1_, fewer child problem behaviors, and a trend toward less physical and psychological discipline. Effects that endured to, or emerged at, *t*_2_ were particularly among observed behaviors and therefore less subject to self‐report biases. Although this must be balanced against the fact that there were also large changes in the control group and so groups were not different in terms of self‐reported harsh discipline or child conduct problems at *t*_2_, the program demonstrates potential for increasing positive parenting. If this effect is strengthened, enduring reductions in child conduct problems may follow.

Among secondary outcomes, *t*_1_ data also revealed a possible, if small, effect on caregiver depression, although this did not maintain to *t*_2_, a pattern found in some other trials (Barlow, Smailagic, Huband, Roloff, & Bennett, 2012). There was also a slight trend toward an increase in parenting stress at *t*_1_ which did not maintain at *t*_2_. It may be that initially caregivers found it a little stressful to remember to use new skills instead of the presumably well‐practiced harsh parenting, or that the training gave them greater insight into the importance of parenting, but that over time they became accustomed to new skills and perspectives; it is also possible that the measure was not stable in the South African context.

Differences between the intervention and control groups were, in general, small, and there were no differences on several variables. Something external to the trial may have caused changes in both groups, but there may be a number of other reasons for this pattern of results. While fidelity to content was maintained, fidelity to process might need improvement: Therapist skill in collaborative processes plays a key role in program effectiveness (Eames et al., 2009), but is harder to learn than content. It is encouraging that paraprofessionals (with only high school education) in a highly deprived community in Africa can successfully deliver a complex group‐based program. Future studies should explore under what conditions paraprofessionals can maintain fidelity to process. For instance, we consider weekly video supervision to be essential, to monitor fidelity and provide feedback and ongoing training (Axford et al., 2017); in future, these session videotapes could be coded to assess facilitator collaborative process skill learning.

Caregiver engagement is another area that may need attention. While much attention is paid to engaging families in high‐income contexts (e.g., Axford et al., 2017), conditions in LMIC are different. In this trial, to mimic conditions that would prevail in program delivery in South Africa, we provided food and a small transport reimbursement, but not child care. Anecdotally, facilitators reported that caregiver alcohol abuse and winter weather inhibited attendance. Despite offering the program on Saturdays, in this context of limited, precarious jobs, we struggled to retain caregivers who gained employment. Future studies should explore reasons for nonengagement and ways to address them that are suitable for LMIC. For instance, embedding the program into existing systems with incentives for participation (such as cash transfers conditioned on attendance), in places of employment, providing other services to parents or providing more material support for attendance (e.g., transport and child care), may enhance attendance and thus effectiveness. We are exploring these possibilities in trials in the Philippines and Thailand, as well as in a factorial experiment in Eastern Europe.

Caregivers faced numerous adversities. It may be that under these conditions, a 12‐session group‐based parenting program is too short to maintain higher levels of positive parenting and establish reductions in child conduct problems without additional support for other challenges. Future studies should investigate means of enhancing the program, for instance by adding material for dealing with IPV.

The program was designed to be as low‐cost as possible. While some costs for any parenting program are unavoidable (e.g., paying facilitators, venue hire), we estimate that as part of routine service delivery costs of delivery may be as low as USD17 per family; training and supervising 20 facilitators are costed at USD20,000. Costs, however, vary depending on local rates of pay, numbers of families per group, and the number of times that trained facilitators deliver the program, and so on.

There are limitations to the trial. One possible reason for the small differences between intervention and control groups is that most measures used have not been validated in South Africa, although the internal consistency findings argue against this as problematic. Furthermore, there may be cultural variations in the way measures are understood: For instance, cultural understandings of parenting may have made the ECBI less sensitive to change in this context and thus unable to detect actual changes in child behavior; similarly, cultural expressions of depression may have meant that the BDI was not sufficiently sensitive. In addition, in the dense living environments of informal settlements, it is possible that there was contamination between intervention and control groups. Future trials should explore whether there is informal dissemination of program content and, if so, consider a cluster trial design. Further, there may have been a testing effect (Shadish, Cook, & Campbell, 2002): Repeated questioning about positive parenting techniques may have either caused a change in parenting itself or elicited stronger social desirability over time; a Solomon four‐square design would be required to rule this out. The changes in the control group suggest that a testing effect, or some other variable external to the study, may have influenced parenting or child behavior, or both.

## Conclusion

There are many strengths to this trial: It was carried out in extremely resource‐poor areas; local paraprofessionals were trained to deliver the program; the recruitment target was exceeded, ensuring sufficient trial power; participants were followed for a year after the program ended with high follow‐up rates; and observational assessments were used to supplement caregiver self‐report. This was a stringent test of PLH for Children and shows that it holds promise as an intervention to support caregivers to learn nonviolent, positive parenting, and, with strengthening, potential for addressing child conduct problems. What remains is to examine how to strengthen that promise, given high need for such programs and demand from policy‐makers (WHO, 2016).

**Key points**

- Parenting programs have been identified as an effective means of reducing violence against children and children's later violence, yet few existing evidence‐based programs are suitable for LMIC contexts.
- We tested PLH for Young Children in a randomized trial in Cape Town, South Africa, with one‐year follow‐up.
- The program shows promise for improving parenting behaviors and reducing child behavior problems and therefore for meeting the SDGs. Further research is urgently needed on the conditions under which programs delivered by paraprofessionals in LMIC can be strengthened.

## Supporting information


**Appendix S1.** Internal consistency of measures.

## References

[jcpp13129-bib-0001] Abidin, R.R. (1997). Parenting Stress Index: A measure of the parent‐child system In Zalaquett C.P. & Wood R.J. (Eds.), Evaluating stress: A book of resources (pp. 277–291). Lanham, MD: Scarecrow Education.

[jcpp13129-bib-0002] Axford, N. , Bywater, T. , Blower, S. , Berry, V. , Baker, V. , & Morpeth, L. (2017). Critical factors in the successful implementation of evidence‐based parenting programmes: Fidelity, adaptation and promoting quality In Dixon L., Perkins D., Hamilton‐Giachritsis C. & Craig L. (Eds.), Critical factors in the successful implementation of evidence‐based parenting programmes (pp. 349–366). Hoboken, NJ: Wiley.

[jcpp13129-bib-0003] Barlow, J. , Smailagic, N. , Huband, N. , Roloff, V. , & Bennett, C. (2012). Group‐based parent training programmes for improving parental psychosocial health. Cochrane Database of Systematic Reviews, (6), CD002020. 22696327 10.1002/14651858.CD002020.pub3

[jcpp13129-bib-0004] Beck, A. , & Steer, R. (1988). Psychometric properties of the Beck Depression Inventory: Twenty‐five years of evaluations. Clinical Psychology Review, 8, 77–100.

[jcpp13129-bib-0005] Chacko, A. , Jensen, S. , Lowry, L. , Cornwell, M. , Chimklis, A. , Chan, E. , … & Pulgarin, B. (2016). Engagement in behavioral parent training: Review of the literature and implications for practice. Clinical Child and Family Psychology review, 19, 204–215. 27311693 10.1007/s10567-016-0205-2

[jcpp13129-bib-0006] Chen, M. , & Chan, K. (2015). Effects of parenting programs on child maltreatment prevention: A meta‐analysis. Trauma, Violence and Abuse, 17, 88–104. 10.1177/1524838014566718 25573846

[jcpp13129-bib-0007] Dunne, M. , Zolotor, A.J. , Runyan, D. , Andreva‐Miller, I. , Choo, W. , Dunne, S.K. , … & Youssef, R. (2009). ISPCAN Child Abuse Screening Tools Retrospective version (ICAST‐R): Delphi study and field testing in seven countries. Child Abuse and Neglect, 33, 815–825. 19853301 10.1016/j.chiabu.2009.09.005

[jcpp13129-bib-0008] Eames, C. , Daley, D. , Hutchings, J. , Whitaker, C. , Jones, J. , & Bywater, T. (2009). Treatment fidelity ASA predictor of behaviour change in parents attending group‐based parent training. Child: Care, Health and Development, 35, 603–612. 10.1111/j.1365-2214.2009.00975.x 19508317

[jcpp13129-bib-0009] Essau, C. , Sasagawa, S. , & Frick, P.J. (2006). Psychometric properties of the Alabama Parenting Questionnaire. Journal of Child and Family Studies, 15, 595–614.

[jcpp13129-bib-0010] Eyberg, S. , & Ross, A. (1978). Assessment of child behavior problems: The validation of a new inventory. Journal of Clinical Child Psychology, 7, 113–116.

[jcpp13129-bib-0011] Furlong, M. , McGilloway, S. , Bywater, T. , Hutchings, J. , Smith, S. , & Donnelly, M. (2013). Behavioural and cognitive‐behavioural group‐based parenting programmes for early‐onset conduct problems in children aged 3 to 12 years. Evidence‐Based Child Health: A Cochrane Review Journal, 8, 318–692. 23877886 10.1002/ebch.1905

[jcpp13129-bib-0012] Gottfredson, D. , Cook, T. , Gardner, F. , Gorman‐Smith, D. , Howe, G. , Sandler, I. , & Zafft, K. (2015). Standards of evidence for efficacy, effectiveness, and scale‐up research in prevention science: Next generation. Prevention Science, 16, 893–926. 25846268 10.1007/s11121-015-0555-x PMC4579256

[jcpp13129-bib-0013] Hillis, S. , Mercy, J. , Amobi, A. , & Kress, H. (2016). Global prevalence of past‐year violence against children: A systematic review and minimum estimates. Pediatrics, 137, e20154079. 26810785 10.1542/peds.2015-4079 PMC6496958

[jcpp13129-bib-0014] Hutchings, J. (2013). The little parent handbook. Bangor, Wales, UK: Children's Early Intervention Trust.

[jcpp13129-bib-0015] Hutchings, J. , Gardner, F. , & Lane, E. (2004). Making evidence‐based intervention work In Farrington D., Sutton C. & Utting D. (Eds.), Support from the start: Working with young children and their families to reduce the risks of crime and antisocial behaviour (pp. 69–79). London: Department for Education and Skills.

[jcpp13129-bib-0016] Knerr, W. , Gardner, F. , & Cluver, L. (2013). Reducing harsh and abusive parenting and increasing positive parenting in low‐ and middle‐income countries: A systematic review. Prevention Science, 14, 352–363. 23315023 10.1007/s11121-012-0314-1

[jcpp13129-bib-0017] Labadarios, D. , Steyn, N. , Maunder, E. , MacIntyre, U. , Gericke, G. , Swart, R. , … & Nel, J.H. (2005). The National Food Consumption Survey (NFCS): South Africa, 1999. Public Health Nutrition, 8, 533–543. 16153334 10.1079/phn2005816

[jcpp13129-bib-0018] Lachman, J. , Cluver, L. , Kelly, J. , Ward, C.L. , Hutchings, J. , & Gardner, F. (2016). Process evaluation of a parenting program for low‐income families in South Africa. Research on Social Work Practice, 282, 188–202.

[jcpp13129-bib-0019] Lachman, J. , Cluver, L. , Ward, C. , Hutchings, J. , Wessels, I. , Mlotshwa, S. , & Gardner, F. (2017). Randomized controlled trial of a parenting program to reduce the risk of child maltreatment in South Africa. Child Abuse and Neglect, 72, 338–351. 28881303 10.1016/j.chiabu.2017.08.014

[jcpp13129-bib-0020] Lachman, J. , Sherr, L. , Cluver, L. , Ward, C.L. , Hutchings, J. , & Gardner, F. (2016). Integrating evidence and context to develop a parenting program for low‐income families in South Africa. Journal of Child and Family Studies, 25, 2337–2352.

[jcpp13129-bib-0021] Leijten, P. , Melendez‐Torres, G.J. , Knerr, W. , & Gardner, F. (2016). Transported versus homegrown parenting interventionsfor reducing disruptive child behavior: A multilevel meta‐regression study. Journal of the American Academy of Child and Adolescent Psychiatry, 55, 610–617. 27343888 10.1016/j.jaac.2016.05.003

[jcpp13129-bib-0022] McEachern, A. , Dishion, T. , Weaver, C. , Shaw, D. , Wilson, M. , & Gardner, F. (2011). Parenting Young Children (PARYC): Validation of a self‐report Parenting Measure. Journal of Child and Family Studies, 21, 498–511. 10.1007/s10826-011-9503-y PMC3412343 22876108

[jcpp13129-bib-0023] Mertens, J. , Ward, C. , Bresick, G. , Broder, T. , & Weisner, C. (2014). Effectiveness of nurse‐practitioner‐delivered brief motivational intervention for young adult alcohol and drug use in primary care in South Africa: A randomized clinical trial. Alcohol and Alcoholism, 49, 430–438. 24899076 10.1093/alcalc/agu030 PMC4060738

[jcpp13129-bib-0024] Mikton, C. (2012). Two challenges to importing evidence‐based child maltreatment prevention programs developed in high‐income countries to low‐ and middle‐income countries: Generalizability and affordability In Dubowitz H. (Ed.), World perspectives on child abuse (Vol. 10, pp. 97). Aurora, CO: ISPCAN.

[jcpp13129-bib-0025] Piquero, A.R. , Farrington, D. , Welsh, B. , Tremblay, R. , & Jennings, W. (2009). Effects of early family/parent training programs on antisocial behavior and delinquency. Journal of Experimental Criminology, 5, 83–120.

[jcpp13129-bib-0026] R Core Team . (2017). R: A language and environment for statistical computing. Vienna, Austria: R Foundation for Statistical Computing Retrieved from https://www.R-project.org

[jcpp13129-bib-0027] Robinson, E. , & Eyberg, S. (1981). The Dyadic Parent‐Child Interaction Coding System: Standardization and Validation. Journal of Consulting and Clinical Psychology, 49, 245–250. 7217491 10.1037//0022-006x.49.2.245

[jcpp13129-bib-0028] Runyan, D. , Dunne, M. , Zolotor, A. , Madrid, B. , Jain, D. , Gerbaka, B. , … & Youssef, R. (2009). The development and piloting of the ISPCAN Child Abuse Screening Tool ‐ Parent version (ICAST‐P). Child Abuse and Neglect, 33, 826–832. 19854511 10.1016/j.chiabu.2009.09.006

[jcpp13129-bib-0029] Shadish, W. , Cook, T. , & Campbell, D. (2002). Experimental and quasi‐experimental designs: For generalized casual inference. Boston, MA: Houghton Mifflin.

[jcpp13129-bib-0030] Sherbourne, C. , & Stewart, A. (1991). The Medical Outcomes Survey (MOS) Social Support Survey. Social Science and Medicine, 32, 705–714. 2035047 10.1016/0277-9536(91)90150-b

[jcpp13129-bib-0031] Straus, M.A. , Hamby, S. , Boney‐McCoy, S. , & Sugarman, D. (1996). The Revised Conflict Tactics Scales (CTS2): Development and preliminary psychometric data. Journal of Family Issues, 17, 283–316.

[jcpp13129-bib-0032] Trizano‐Hermoslia, I. , & Alvarago, J. (2016). Best alternatives to Cronbach's alpha reliability in realistic conditions: Congeneric and asymmetrical measurements. Frontiers in Psychology, 7, 769. 27303333 10.3389/fpsyg.2016.00769 PMC4880791

[jcpp13129-bib-0033] WHO (2016). INSPIRE: Seven strategies for ending violence against children. Geneva, Switzerland: WHO.

