# Supplementary material for: Parenting for Lifelong Health for Young Children: a randomized controlled trial of a parenting program in South Africa to prevent harsh parenting and child conduct problems
Source: J Child Psychol Psychiatry. 2019 Sep 19;61(4):503–12. doi: 10.1111/jcpp.13129 (PMC7155004; doi:10.1111/jcpp.13129)
Supplement: Supplementary file 1 — Appendix S1. Internal consistency of measures. [file JCPP-61-503-s001.docx]

# Appendix S1

Parenting for Lifelong Health Young Children: A randomized controlled trial of a parent skills training program to prevent harsh parenting and child conduct problems

Catherine L. Ward, Inge M. Wessels, Jamie M. Lachman, Judy Hutchings, Lucie D. Cluver, Reshma Kassanjee, Raymond Nhapi, Francesca Little, and Frances Gardner

|  | | |  |
| --- | --- | --- | --- |
|  | Cronbach’s alpha | Coefficient omega | Greatest Lower Bound |
|  | a [CI] | ω [CI] | GLB |
| Eyberg Child Behavior Inventory - intensity | 0.89 [0.87, 0.91] | 0.90 [0.88, 0.91] | 0.93 |
| Eyberg Child Behaviour Inventory – problem | 0.89 [0.86, 0.90] | 0.90 [0.87, 0.90] | 0.92 |
| Positive Parenting – frequency | 0.7575 [0.72, 0.80] | 0.76 [0.72, 0.80] | 0.85 |
| Positive Parenting – problem | 0.8412 [0.82, 0.87] | 0.85 [0.83, 0.88] | 0.86 |
| Non-Violent Discipline | 0.2897 [0.16, 0.41] | 0.46 [0.42, 0.53] | 0.47 |
| Physical Discipline | 0.5808 [0.50, 0.66] | 0.61 [0.54, 0.68] | 0.63 |
| Psychological Discipline | 0.6321 [0.58, 0.69] | 0.64 [0.57, 0.70] | 0.77 |
| Beck Depression Inventory | 0.8992 [0.88, 0.91] | 0.90 [0.88, 0.92] | 0.94 |
| Parenting Stress | 0.9093 [0.89, 0.92] | 0.91 [0.89, 0.92] | 0.90 |
| Parent Social Support | 0.8515 [0.83, 0.88] | 0.86 [0.83, 0.88] | 0.89 |
